# Evolutionarily Conserved Pattern of AMPA Receptor Subunit Glycosylation in Mammalian Frontal Cortex

**DOI:** 10.1371/journal.pone.0094255

**Published:** 2014-04-08

**Authors:** Janusz Tucholski, Anita L. Pinner, Micah S. Simmons, James H. Meador-Woodruff

**Affiliations:** Department of Psychiatry and Behavioral Neurobiology, University of Alabama at Birmingham, Birmingham, Alabama, United States of America; Indian Institute of science, India

## Abstract

Protein glycosylation may contribute to the evolution of mammalian brain complexity by adapting excitatory neurotransmission in response to environmental and social cues. Balanced excitatory synaptic transmission is primarily mediated by glutamatergic neurotransmission. Previous studies have found that subunits of the AMPA subtype of glutamate receptor are N-glycosylated, which may play a critical role in AMPA receptor trafficking and function at the cell membrane. Studies have predominantly used rodent models to address altered glycosylation in human pathological conditions. Given the rate of mammalian brain evolution and the predicted rate of change in the brain-specific glycoproteome, we asked if there are species-specific changes in glycoprotein expression, focusing on the AMPA receptor. N-glycosylation of AMPA receptor subunits was investigated in rat (*Rattus norvegicus*), tree shrew (*Tupaia glis belangeri*), macaque (*Macaca nemestrina)*, and human frontal cortex tissue using a combination of enzymatic deglycosylation and Western blot analysis, as well as lectin binding assays. We found that two AMPA receptor subunits, GluA2 and GluA4, are sensitive to deglycosylation with Endo H and PNGase F. When we enriched for glycosylated proteins using lectin binding assays, we found that all four AMPA receptor subunits are glycosylated, and were predominantly recognized by lectins that bind to glucose or mannose, N-acetylglucosamine (GlcNAc), or 1-6αfucose. We found differences in glycosylation between different subunits, as well as modest differences in glycosylation of homologous subunits between different species.

## Introduction

Protein glycosylation regulates a wide range of processes critical to development and functioning of the central nervous system, including cell adhesion, cellular migration and differentiation, as well as synaptogenesis, synaptic efficacy and plasticity. The importance of glycosylation in the brain is underscored by the detrimental effects of impaired synthesis of glycoconjugates found in glycosylation congenital disorders (GCDs), including developmental delays, progressive atrophy, psychomotor deficits, seizures, and strokes or stroke-like symptoms [1]. The majority of brain glycoproteins are predicted to be N-glycosylated [2]. In addition, proteins of the mammalian brain have the highest number of predicted N-glycosylation sites and the highest number of tissue specific N-glycosylated proteins [3], suggesting that brain-specific N-glycoproteins coevolved synergistically with its increasing anatomical and functional complexity.

The ability to adapt and respond to environmental cues coevolved with the complexity of the mammalian brain, and depends on balanced excitatory synaptic transmission. Glutamate is the major neurotransmitter involved in fast excitatory transmission, which is primarily mediated by the AMPA subtype of glutamate receptor [4], [5]. AMPA receptors are responsible for postsynaptic depolarization, conveying fast ‘point-to-point’ signaling of neurons [6]. The AMPA receptor subunits are N-glycosylated after their biosynthesis in the lumen of the endoplasmic reticulum (ER) [7]–[10], yet the functional role of this posttranslational modification is not well understood [11]. N-glycosylation has no intrinsic effects on ligand binding or ion receptor conductivity, as recombinant receptors without N-glycans attached can still function as ion channels [8], [12]. However, N-glycosylation may play an important role in the trafficking and stabilization of AMPA receptors at the synapse. For example, we have previously reported that there is a smaller population of GluA2 attached to N-linked high mannose containing glycans in dorsolateral prefrontal cortex in patients with schizophrenia, which we interpreted as consistent with accelerated forward trafficking of the GluA2-containing AMPA receptors [13]. Glycosylation may also affect neurodevelopment: GluA2 in mouse hippocampus expresses the human natural killer-1 (HNK-1) glycol-epitope, which may be essential for dendritic spine morphogenesis in developing neurons [14], [15].

Studies aimed at understanding the function of mammalian brain have predominantly used rodent models. However, given the significant evolutionary distance between rodents and humans, it remains unclear to what extent data from rodent studies can be used to understand human disorders associated with abnormalities of glutamate neurotransmission. In the current study, we asked if one can use findings from animal models to uncover roles that glycans/glycoproteins may play in normal brain and begin to address dysfunction of glycosylation in pathological conditions, given the rapid rate of human brain evolution and the estimated rate of change in the brain- specific glycoproteome [16]. To that end, we compared N-glycosylation in brain of GluA1-4 between four mammalian species (rat, tree shrew, macaque, and human), with the hypothesis that we would observe evolutionarily distinct patterns of glycosylation of AMPA receptors, which in turn might reflect intrinsic differences in biosynthesis, processing, trafficking, or interaction of the receptor subunits with cellular and extracellular partners.

## Materials and Methods

### Ethics statement

Human brain samples were obtained in accordance with the UAB Institutional Review Board and in accordance with The Code of Ethics of the World Medical Association. All tissue donations were obtained through the Alabama Organ Center in compliance with the Alabama uniform anatomical gift act. Samples were deidentified prior to being required by us for these experiments. Macaques (*Macaca nemestrina*) brain samples were obtained from the Regional Primate Center of the University of Washington. Macaques were sacrificed as part of projects unrelated to the present study, which did not require brain tissue. No information was available to us about the method of sacrifice or treatment of macaques in the original studies. Protocols involving these animals were reviewed by the Washington Regional Primate Research Review Committee and by the University of Washington Institutional Animal Care and Use Committee (IACUC). Rat (*Rattus norvegicus*) and tree shrew (*Tupaia glis belangeri*) studies and procedures were performed according to UAB guidelines and approved by the UAB IACUC.

### Tissue acquisition and preparation

Samples of frontal cortex from four mammalian species were used for this study. Human tissue samples were provided by Dr. Rosalinda Roberts and the Alabama Brain Collection. Tree shrew samples were provided by Dr. Thomas Norton at the University of Alabama at Birmingham, and rats were provided by Dr. Lori McMahon at UAB. For this study samples were obtained from three unique subjects of each species, and stored at −80°C until use. Cortical blocks from each subset were homogenized by hand with a glass dounce homogenizer (Bellco Glass, Vineland, NJ, USA) in ice-cold buffer [5 mM Tris-HCl (pH 7.5), 0.32 M sucrose] with a protease inhibitor tablet (Complete Mini, EDTA-free; Roche Diagnostics, Mannheim Germany). Protein concentration was determined using bicinchoninic acid (BCA) protein assay kits (Thermo Scientific, Rockford, Illinois). Homogenates were stored at −80°C until used for specific experiments.

### Enzymatic deglycosylation of AMPA subunits

Samples (50 μg total protein per reaction) were incubated at 37°C for 16 hours with two different glycosidases, endoglycosidase H (Endo H) or peptide-N-glycosidase F (PNGase F) (QA-Bio, Palm Desert, CA) as we have previously described [13], [17]. Proteins were separated using 4–12% Bis-Tris gradient SDS-polyacrylamide gel electrophoresis (SDS-PAGE) (Invitrogen, Carlsbad, CA), transferred to nitrocellulose membranes, and probed with the following primary antibodies: anti-GluA1 rabbit polyclonal (Millipore, Bellarica, CA, # 07-660; 1∶1000), anti-GluA2 mouse monoclonal (US Biological, Swampscott, MA, # G3500; 1∶6000), anti-GluA3 (Millipore, # MAB5416; 1∶1000), or anti-GluA4 rabbit polyclonal (Millipore, # 06-308; 1∶1000). All antibodies were previously optimized for each protein to determine optimal conditions within the linear range of detection for each assay, and that the primary antibody was present in excess. The membranes were washed in Tris-buffered Saline +0.05% Tween-20 (TBST) and probed with IR-dye labeled secondary anti-mouse or anti-rabbit antibodies (LiCor, Lincoln, NE; 1∶10 000) for 1 hour at room temperature (RT). The membranes were rinsed five times for 5 minutes with TBST and briefly with MiliQ water before analyzed [13].

### Lectin binding assays

Lysates prepared from frontal cortex were first solubilized with 1% Triton X-100 and 1% SDS to dissociate AMPA receptor subunits. For each assay, 400 μg lysate was incubated with 20 μg lectin (Vector Laboratories, Burlingame, CA), rotating for 16 hours at 4°C. Streptavidin M-280 Dynabeads (Life Technologies, Grand Island, NY) were washed with TBST before adding 70 μl to each reaction. The samples were then incubated with the beads, rotating for 2.5 hours at RT. The beads were rinsed with TBST, and bound protein was eluted from the beads with 2X sample buffer for 10 minutes at 70°C. To measure individual AMPA receptor subunits bound to each lectin, Western blotting was performed as described above. Blots were probed with the following primary antibodies: anti-GluA1 rabbit polyclonal (Millipore, Billerica, MA, #AB1504, 1∶100); anti-GluA2 mouse monoclonal (US Biological, Swampscott, MA, #G3500, 1∶1000); anti-GluA3 mouse monoclonal, (Millipore, #MAB5416, 1∶250); or anti-GluA4 rabbit polyclonal (Cell Signaling, Boston, MA#8070 XP, 1∶250).

### Immunoprecipitation

To compare glycosylation status of individual subunits, we first ensured dissociation of the subunits from their native AMPA receptor complex. Given the AMPA receptor composition of subunits with hydrophobic transmembrane domains, and the inherent propensity to co-immunoprecipitate associated proteins, we determined optimal solubilization conditions to isolate each individual AMPA receptor subunit. Past studies suggest that a predominance of AMPA receptors contain the GluA2 subunit [18]. Accordingly, we optimized the immunoprecipitation of GluA2 with several detergents and used Western blot analysis for GluA1, GluA3, and GluA4 to confirm subunit dissociation.

Cortical homogenates were solubilized at 4°C with rotation for 16 hours. Lysates were centrifuged at 2300 x g at 4°C for 10 minutes. Supernatants were removed and protein concentrations were determined using BCA protein assay kits. For each GluA2 immunoprecipitation, 50 μl of M-280 sheep anti-Mouse IgG Dynabeads (Life Technologies, Grand Island, NY) were washed with cold TBST before incubating 15 minutes with 5 μg anti-GluA2 antibody (United States Biological, Salem, MA, # G3500-13G) at RT with rotation. Excess antibody was removed with a TBST rinse, and the antibody-bound beads were incubated with 400 μg lysates from human frontal cortex by rotating 30 minutes at RT. The beads were washed with TBST and bound protein eluted with 2X sample buffer for 10 minutes at 70°C. Lysis buffer containing 1% Triton X-100 and 1% SDS was found to be optimal to fully dissociate AMPA receptor subunits before immunoprecipitation.

### Data analysis

The near-infrared fluorescence values for each protein band corresponding to AMPA subunit proteins were collected using a LiCor Odyssey scanner and were expressed as raw integrated intensity with top-bottom median intralane background subtraction using Odyssey V3.0 analytical software [13], [19].

**Figure 1:**
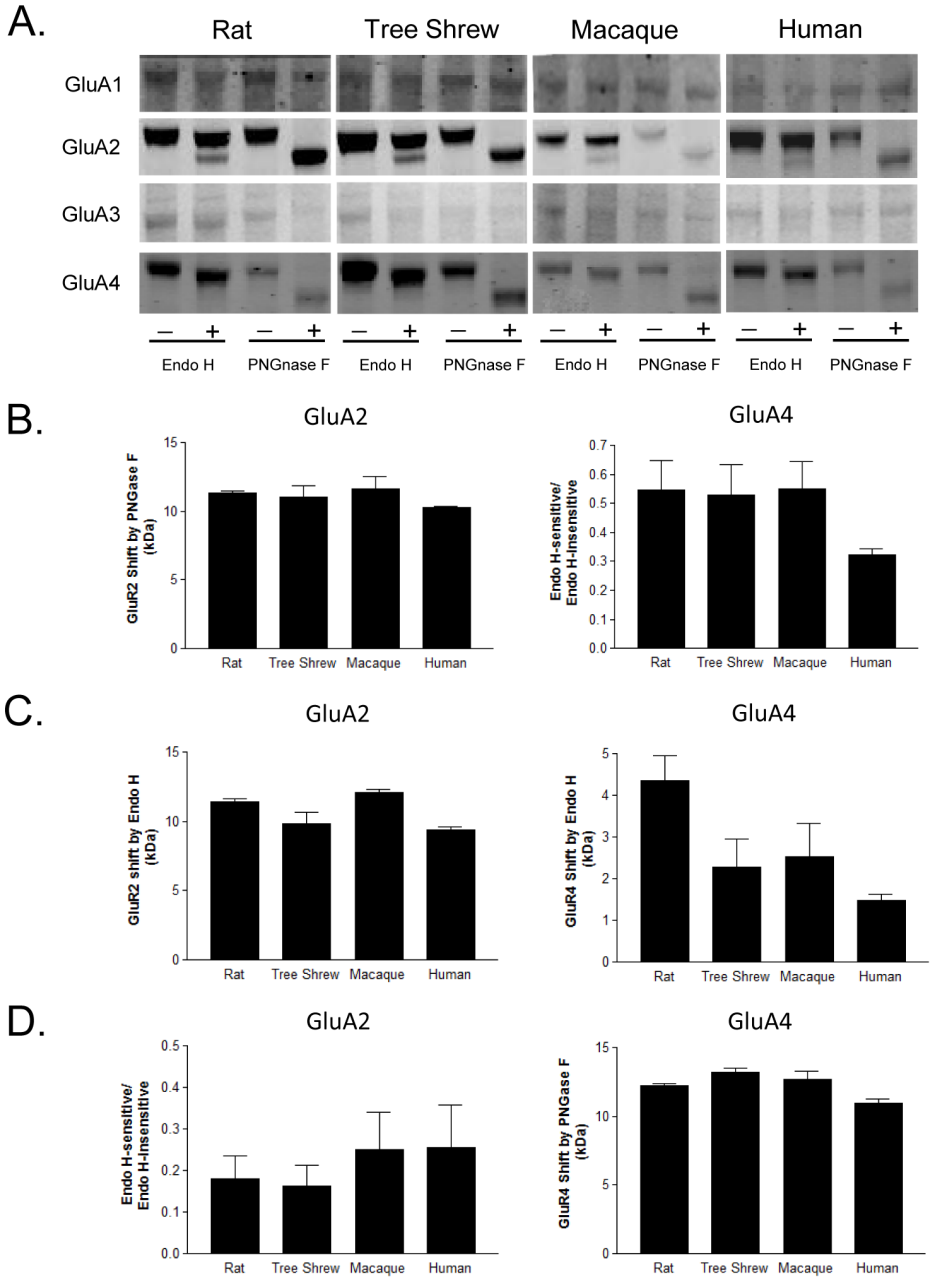
Enzymatic deglycosylation assay of AMPA receptor subunits. (A) Representative immunoblots showing changes in electrophoretic mobility for GluA2 and GluA4, but not GluA1 or GluA3, following deglycosylation with Endo H or PNGase F in rat, tree shrew, macaque, and human frontal cortex. (B) Quantification of Endo H-sensitive molecular mass shifts for GluA2 and GluA4. Data are presented as the distance between the Endo H-deglycosylated protein band of higher mobility, and the same protein without Endo H treatment. (C) Quantification of Endo H-sensitive molecular mass shifts for GluA2 and GluA4. Graphs show the ratio of measured intensities of the Endo H-sensitive band to the Endo H-insensitive band. (D) Quantification of PNGase F-sensitive molecular mass shifts for GluA2 and GluA4. Data are presented as the distance between the PNGase F deglycosylated protein band of higher mobility, and the same protein without PNGase F treatment. Graphs represent means ± SD calculated from three independent experiments.

**Figure 2:**
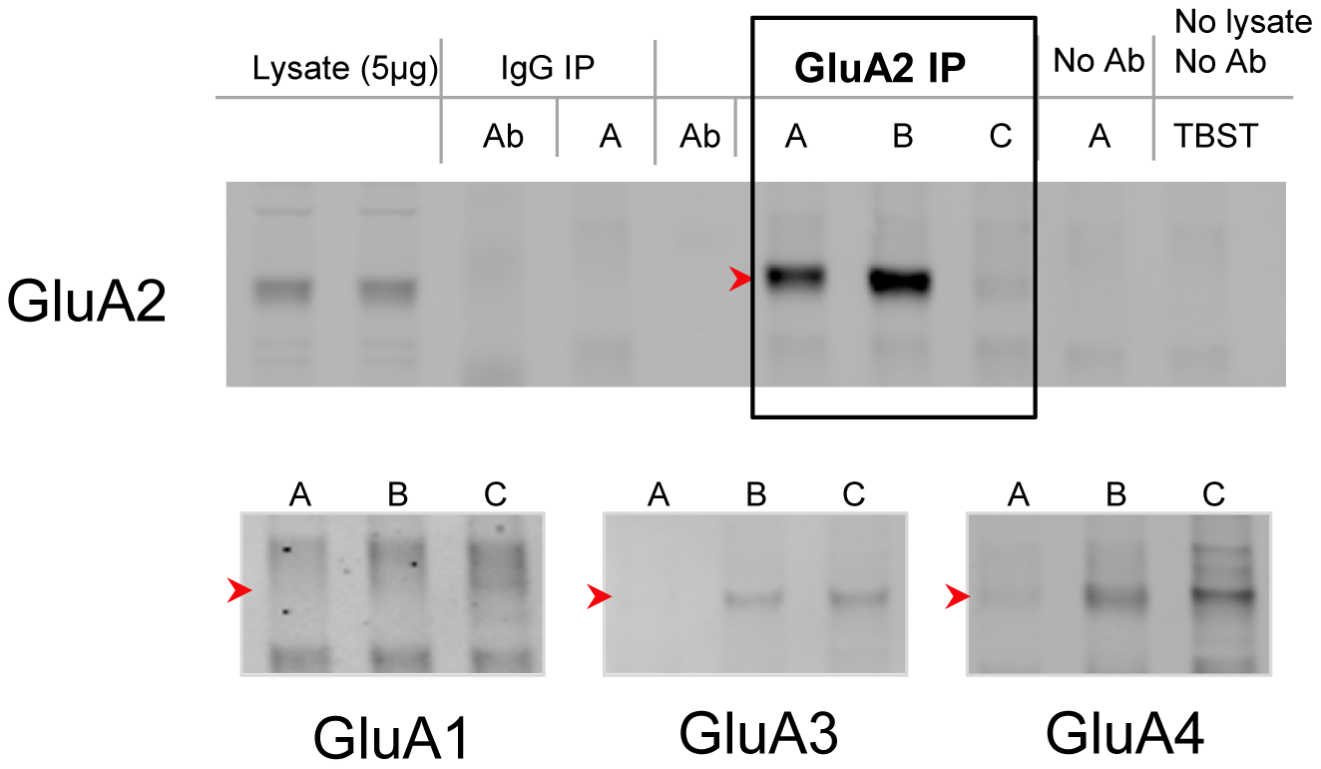
Effective immunoprecipitation of AMPA receptor subunits from human frontal cortex. GluA2 was immunoprecipitated from homogenized human frontal cortex in buffers with (A) 1% Triton X-100 plus 1% SDS, (B) 1% Triton X-100 plus 0.5% SDS, or (C) 1% Triton X-100. GluA2 was immunoisolated without co-precipitation of the GluA1, GluA3, or GluA4 subunits only in conditions with 1% SDS.

**Figure 3:**
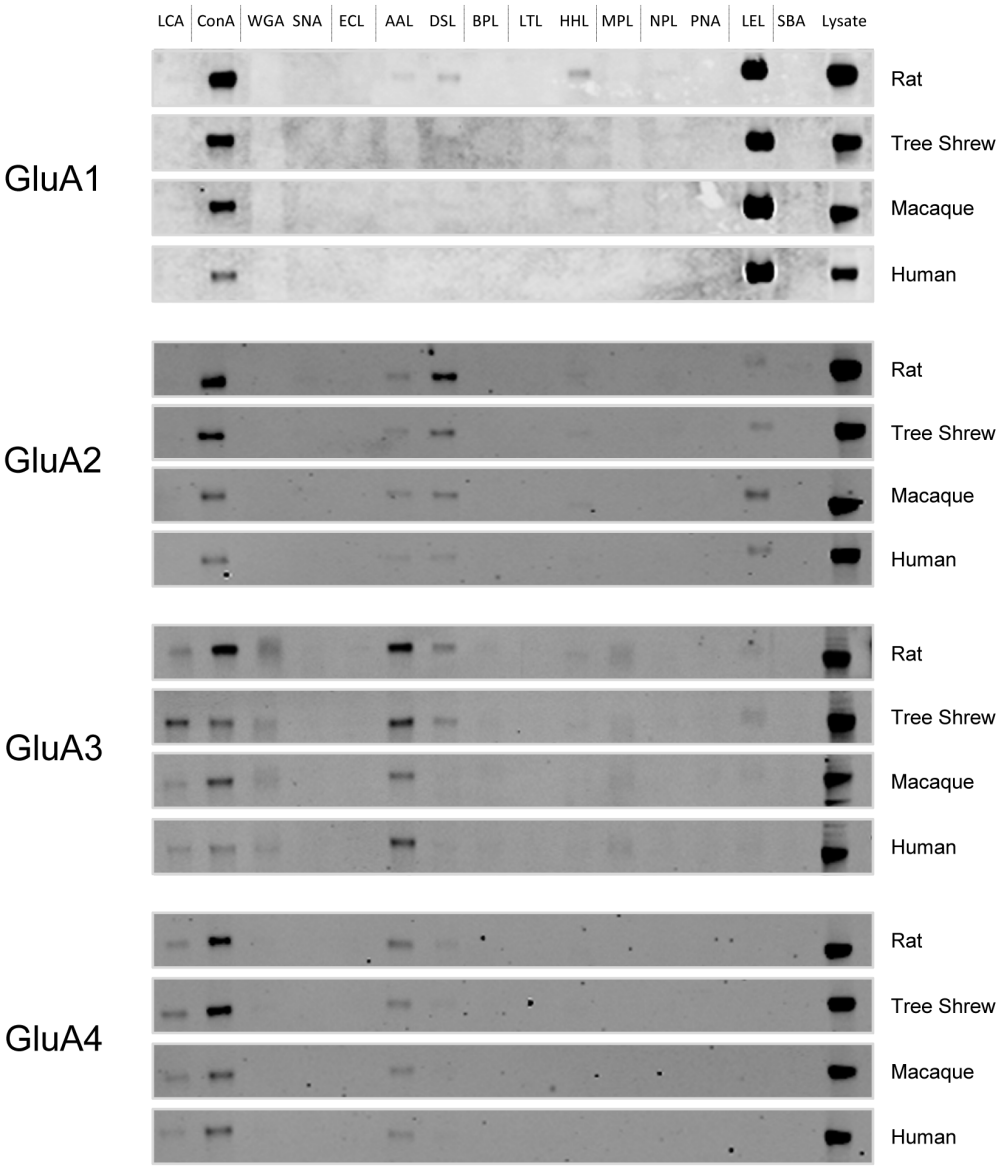
AMPA receptor subunit binding to lectins. AMPA receptor subunits from frontal cortex of rat, tree shrew, macaque and human cortex were purified with 15 biotinylated lectins (Figure 5). Proteins that bound to lectins were separated by SDS–polyacrylamide electrophoresis, transferred to nitrocellulose membranes, and probed using specific antisera for each AMPA receptor subunit.

Given that we found that only GluA2 and GluA4 were sensitive to Endo H and PNGase F treatment, we analyzed N-glycosylation of these two AMPA receptor subunits after enzymatic deglycosylation by quantifying three dependent variables: (1) Endo H-sensitivity was quantified for each subject as the ratio of intensities of Endo H-sensitive to Endo H-insensitive bands (Figure 1B); (2) Endo H-sensitivity was also quantified by measuring the molecular mass shifts for GluA2 and GluA4 after Endo H treatment (Figure 1C); (3) PNGase F-sensitive molecular mass shifts were quantified by measuring the distance between PNGase F deglycosylated protein bands of higher mobility and the same protein without PNGase F treatment (Figure 1D).

**Figure 4:**
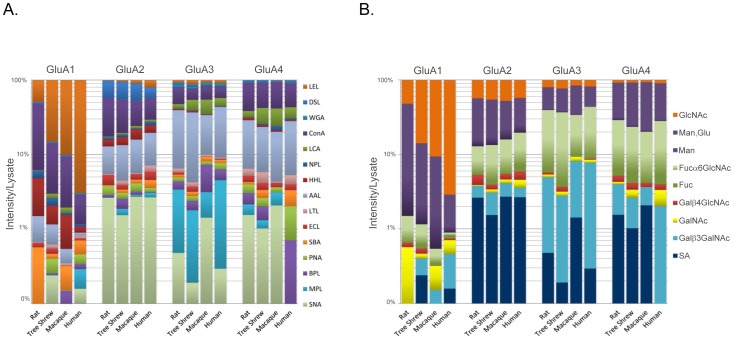
Glycosylation of AMPA receptor subunits as percentage of total subunit expression. Bar graphs represent the percentage (in log scale) that each lectin (Figure 4A) or sugar (Figure 4B) that lectins recognize contributes to total subunit expression, as detected by this panel of lectins (Figure 5), for each species.

**Figure 5:**
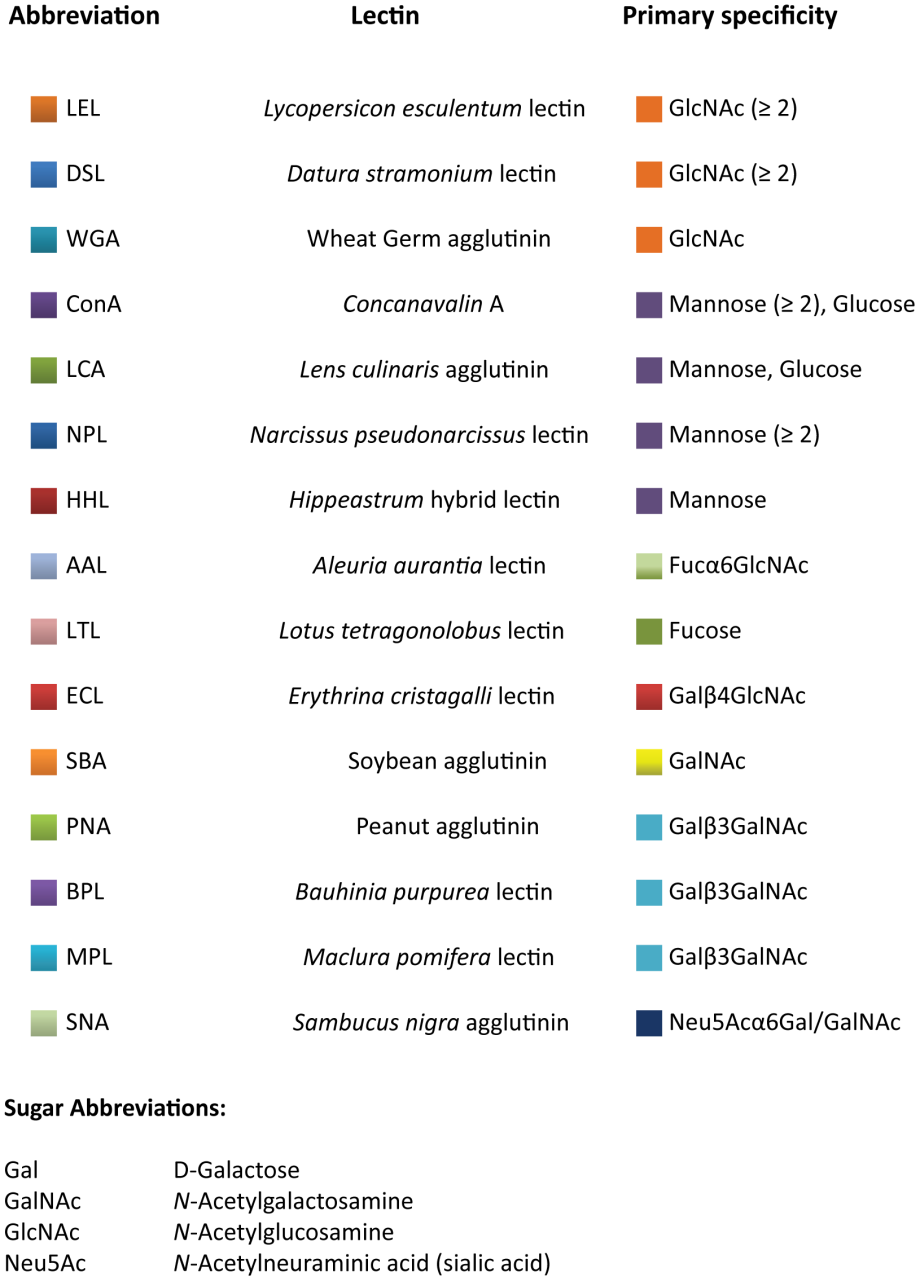
Glycan specificity of lectins.

Analysis of the glycosylation of individual AMPA receptor subunits (purified at optimal solubilization conditions, Figure 2) as determined from lectin binding was performed by first normalizing the intensity of each band to same-blot lysate lane expression (Figure 3). The resulting bar graphs represent the percentage that each lectin (Figure 4A) or glycan (Figure 4B) contributes to total expression as detected by this panel of lectins (Figure 5), for each subunit across species.

## Results

### AMPA subunits sensitive to enzymatic deglycosylation across species

We investigated the presence and molecular mass of N-glycans attached to each AMPA receptor subunit in rat, tree shrew, macaque, and human frontal cortex using enzymatic deglycosylation assays [13], [17], [20]. The analysis of Endo H or PNGase F treated samples revealed that GluA2 and GluA4 were the only two AMPA receptor subunits sensitive to Endo H or PNGase F-mediated deglycosylation (Figure 1, as we have previously reported in human brain [21]). Endo H treatment resulted in two bands of approximately 98 and 90 kDa (Figure 1). The 90 kDa band was markedly more pronounced for the GluA2 than the GluA4 subunit. PNGase F treatment produced a single band of ∼88 kDa (Figure 1A) for both subunits. The 9–12 kDa shift of both subunits after treatment with PNGase F may indicate the presence of several N-linked glycans, given that an average N-linked glycans has an apparent molecular mass of 1.5 to 3 kDa [22]. For each species, a similar pattern of enzymatic deglycosylation was observed.

We next explored species-specific differences in extent of N-linked glycosylation of the AMPA receptor subunits. We found that a larger population of GluA2 in human and macaque appears more sensitive to deglycosylation by Endo H, suggesting that more GluA2 subunits in these species contain N-linked high-mannose or hybrid sugars. For GluA4, however, these N-linked high-mannose or hybrid sugars were increased in rat, tree shrew, and macaque, but decreased in humans (Figure 1B). When measuring post-deglycosylation molecular mass shifts, rats and macaques had the heaviest mass of sugars attached to GluA2 compared to the other species (Figure 1C). The molecular mass shift of GluA4 was more complex. The rat samples had the heaviest mass of attached sugars, while tree shrew and macaque had lower mass, and human the least amount of attached sugars (Figure 1C). PNGase F treatment, which removes all N-linked glycans, revealed that for both GluA2 and GluA4 that humans have the lowest total mass of N-linked sugars compared to other species (Figure 1D).

No molecular mass shifts were measurable for GluA1 or GluA3, suggesting either lack of significant populations of N-glycosylated forms of these subunits, or the presence of smaller molecular masses of N-linked glycans that cannot be detected by enzymatic deglycosylation shift assays. GluA2 and GluA4 have subtle but diverse differences of N-linked glycans across species, providing evidence of the complexity of N-glycosylation in the brain.

### Lectin-binding assays uncover the complexity of glycans attached to AMPA receptor subunits

Lectin binding assays were used to further characterize the glycans attached to individual AMPA receptor subunits. Each sample was treated with 1% SDS and 1% Triton X-100 to dissociate individual subunits (Figure 2). Then, each AMPA receptor subunit was precipitated with a panel of 15 biotinylated lectins (Figure 5) that bind specifically to mono- or oligosaccharides [23] (Figure 3, 4). The majority of glycans attached to AMPA receptor subunits detected by this panel of lectins contained either glucose or mannose (recognized by ConA and LCA), GlcNAc (recognized by DSL, LEL, and WGA), or fucose (recognized by AAL).

The glycosylation pattern for each subunit was generally conserved across species, with notable exceptions for several glycans. The GlcNAc moiety was strikingly higher for the GluA1 subunit, and markedly increased from rat to human. Binding affinity to GlcNAc decreased from GluA1, which had considerably more binding than GluA2, followed by GluA3 and GluA4 in all four species. Also noteworthy was the reciprocal binding for GlcNAc and mannose/glucose in GluA1; as GlcNAc binding increased from rat to human, it decreased for mannose/glucose binding in that subunit. However, the GlcNAc and mannose/glucose moieties were comparable across species in the other subunits. The Fucα6GlcNAc moiety appeared to be higher in GluA3 than other subunits and comparable across species for subunits GluA2-GluA4.

## Discussion

In this study, we characterized the glycosylation of AMPA receptor subunits in the frontal cortex from four mammalian species (rat, tree shrew, macaque, and human) using Western blot analysis, following enzymatic deglycosylation and by lectin binding assays. As we have previously shown in the human [13], we found that two AMPA receptor subunits, GluA2 and GluA4, are sensitive to deglycosylation with Endo H and PNGase F, consistent with large molecular masses of glycans attached to these subunits. When we enriched for glycosylated proteins using lectin binding assay, we were able to detect glycans attached to all four AMPA receptor subunits. We also noted species-specific patterns of glycosylation, although these were generally modest differences.

Each AMPA receptor subunit was precipitated with a panel of 15 biotinylated lectins, 6 of which were most associated with the GluA subunits (Figure 4A). Given that lectins bind glycan moieties attached to glycoproteins with fairly known specificity [24], we were able to determine the percentage that each lectin, and by inference glycan, contributed to the total expression of glycans attached to each AMPA receptor subunit. These subunits were predominantly recognized by lectins that bind to glucose or mannose (ConA, LCA, HHL), N-acetylglucosamine (GlcNAc) (DSL, LEL, WGA), or 1-6αfucose (AAL). The most striking difference that we found from the lectin studies was the affinity of GluA1 to LEL, a lectin recognizing GlcNAc . GluA1 showed marked affinity to LEL, whereas for GluA4 this affinity was almost negligible. Also noteworthy was the reciprocal binding observed for GluA1 to GlcNAc and mannose/glucose recognizing lectins. As GlcNAc associated binding evolutionarily increased from rat to human, it decreased for mannose/glucose recognizing lectins. It is likely that most of this lectin binding is primarily associated with N-linked glycans, given that all AMPA receptor subunits contain 4 to 6 N-glycosylation cognate sites (Asn-X-Ser/Thr (X ≠ Pro)) [11], [25], and that there is no evidence to date for O-glycosylation of AMPA receptor subunits.

N-linked glycosylation occurs in the ER with subsequent modification in the Golgi apparatus; movement of the AMPA subunits through the ER and Golgi can be inferred by their sensitivity to Endo H. Glycoproteins that contain high mannose and hybrid chains are sensitive to Endo H-driven deglycosylation while they are in the ER and in proximal regions of the Golgi complex. In the mid Golgi apparatus, glycans are modified to more complex structures which become Endo H insensitive. However, all N-linked glycans are sensitive to PNGase F, and only the addition of α1,3fucose in invertebrates and plants has been described to confer resistance to this glycosidase [26], [27].

This study is consistent with our previous findings that in the human frontal cortex, GluA2 and GluA4 are the only subunits sensitive to deglycosylation by Endo H and PNGase F. Analysis of deglycosylation patterns revealed that a larger population of GluA2 in human and macaque contained Endo H-sensitive high mannose or hybrid glycans. This was restricted to GluA2; a smaller population of GluA4 was sensitive to Endo H in human cortex. An interpretation of these current findings is that in macaque and human cortex there is a larger population of GluA2 associated with the ER and/or early Golgi cellular compartments. Interestingly, earlier studies have demonstrated that the ER localization of the GluA2 subunit is essential for assembly of AMPA receptor complexes, the exit of assembled receptors from the ER, and forward trafficking to the synaptic membrane [28], [29]. Not detecting N-linked glycans by deglycosylation assays was somewhat surprising, given previous findings that the GluA1 and GluA3 subunits from rat frontal cortex were sensitive to enzymatic glycosylation [30]. Our data suggest either the possibility of a low molecular mass of N-linked glycans on GluA1 and GluA3 subunits that went undetectable by our enzymatic deglycosylation, or alternatively the presence of an unknown modification that confers glycans resistance to enzymatic cleavage [26]. The lectin binding assay is more sensitive due to the enrichment of glycans specific to each lectin, but is not specific to the type of glycosylation. For example, O-linked glycosylation of AMPA receptor subunits is a possibility that could explain the lack of detection of N-glycosylation by Endo H or PNGase F, and potentially consistent with the presence of glycosylated GluA1 and GluA3 subunits that we found by lectin binding.

The modest differences that we found in glycosylation of AMPA receptor subunits in the human may reflect intrinsic differences in biosynthesis, processing, trafficking, or interaction of the receptor subunits with cellular and extracellular partners. Recent evolutionary findings suggest that within each phylogenetic branch, the extracellular glycoproteome coevolved by adapting to extracellular environmental cues specific to development, growth, and organ formation [16]. Extracellular N-glycosylated proteins appear to have evolved at much faster rate than intracellular glycoproteins, except for extracellular N-glycosylated Asn. Interestingly, the non-N-glycosylated Asn evolved at significantly higher rate than N-glycosylated, suggesting a significant evolutionary pressure to retain N-glycosylated Asn residues [16]. Both the intracellular and extracellular glycoproteomes are essential for cell survival, while balanced excitatory synaptic transmission is necessary for higher functioning. Genetic information for glycosylation is conserved, yet the versatility of post-translational modifications varies among species, notably in the terminal glycans. This suggests that AMPA receptors exit the Golgi after terminal glycosylation driven by species-specific cellular and environmental pressures.

In summary, this study compares the glycosylation pattern of AMPA receptor subunits in the frontal cortex across different mammalian species. We found that all four AMPA receptor subunits are glycosylated, but also demonstrated that there are differences in glycosylation between different subunits as well as modest differences in glycosylation of homologous subunits between different species.
